# Tolerogenic therapies in transplantation

**DOI:** 10.3389/fimmu.2012.00198

**Published:** 2012-07-18

**Authors:** Eugenia K. Page, Wasim A. Dar, Stuart J. Knechtle

**Affiliations:** Department of Surgery, Emory University Hospital,Atlanta, GA, USA

**Keywords:** B cell therapeutics, cellular therapies, costimulation blockade, mixed chimerism, regulatory T cells, T cell depletion, tolerance, transplantation

## Abstract

Since the concept of immunologic tolerance was discovered in the 1940s, the pursuit of tolerance induction in human transplantation has led to a rapid development of pharmacologic and biologic agents. Short-term graft survival remains an all-time high, but successful withdrawal of immunosuppression to achieve operational tolerance rarely occurs outside of liver transplantation. Collaborative efforts through the NIH sponsored Immune Tolerance Network and the European Commission sponsored Reprogramming the Immune System for Establishment of Tolerance consortia have afforded researchers opportunity to evaluate the safety and efficacy of tolerogenic strategies, investigate mechanisms of tolerance, and identify molecular and genetic markers that distinguish the tolerance phenotype. In this article, we review traditional and novel approaches to inducing tolerance for organ transplantation, with an emphasis on their translation into clinical trials.

## INTRODUCTION

Immunologic tolerance was first introduced in 1945 when Ray Owen observed that placental interchange resulted in red cell chimerism between dizygotic bovine twins (Owen, 1945). In the ensuing decade, Peter Medawar, McFarlane Burnet, and colleagues elaborated upon this phenomenon of acquired immunologic tolerance with experimental models of transplantation, which awarded them the Nobel Prize in Physiology or Medicine in 1960. Most of the work at the time involved non-self antigen exposure in immunologically immature hosts, until 1959 when Schwartz and Dameshek demonstrated a marked delay in the adult rabbit immune response to iodine-labeled injections of human serum albumin when treated with 6-mercaptopurine (Schwartz and Dameshek, 1959). Their descriptions of the inhibition of immune pathways in this “drug-induced immunological tolerance” notably foreshadowed the era of pharmacologic development for tolerance induction.

The next 50 years heralded a boom in drug development and subsequent improvements in graft survival. In contrast to 1-year graft survival in 1977 of 53 and 78% for deceased and living-related donors, respectively (Eggers, 1988), modern immunosuppression has enabled transplant recipients to enjoy very favorable graft survival. One-year rates having asymptotically approached 93–96%; therefore, short-term graft survival alone can no longer be held as the metric of success for new immunosuppressants. Instead, as 10-year graft survival rates still trail at 47–61%, new agents must address factors leading to chronic rejection as well as the comorbidities associated with chronic immunosuppression. The decisive measure of success is for a therapy to demonstrate allospecific immunosuppression while minimizing side effects and preserving immune competence to infectious pathogens and cancer during drug administration, and permanent graft survival after its withdrawal.

While transplant tolerance has been largely elusive in human organ transplantation, it has been an achievable feat in animal – particularly murine – models. Non-human primate studies have identified successful preclinical tolerogenic approaches, from T cell depletion and mixed chimerism to costimulation blockade and cellular therapies (Hamawy and Knechtle, 1998; Kawai et al., 2011). Our experience with FN18-CRM9 CD3 immunotoxin in rhesus macaques showed that T cell depletion led to graft survival over 600 days, with five of six long-term survivors demonstrating donor-specific tolerance by skin grafting (Knechtle et al., 1997; Torrealba et al., 2003). Kawai et al. (1995) reported tolerance induction in four cynomolgus macaques that developed multilineage mixed chimerism. Costimulation (CD154) blockade enhanced mixed chimerism and tolerance induction when added to their chimerism-inducing non-myeloablative regimen (Kawai et al., 2004). In the above studies, however, a considerable number of animals developed chronic rejection, sometimes even years before their grafts were terminally rejected. This underscores the metastable nature of tolerance, at least in non-human primates, which is likely mediated by donor-specific regulatory T cells expressing TGFβ (Knechtle and Burlingham, 2004; Torrealba et al., 2004; Ashton-Chess et al., 2007).

Tolerance is infrequently achieved outside of liver transplantation in humans and is often encountered serendipitously due to non-compliance or physician-driven immunosuppression withdrawal for severe adverse effects or malignancy. In clinical practice, operational tolerance is defined as “a well-functioning graft lacking histological signs of rejection, in the absence of any immunosuppressive drugs (for at least 1 year), in an immunocompetent host” (Ashton-Chess et al., 2007; Orlando et al., 2010). Orlando et al. (2009) provided a comprehensive review of all successful and unsuccessful cases of clinical operational tolerance after liver or kidney transplantation. One hundred of 461 liver recipients (22%) remained immunosuppression free 1 year after withdrawal; a total of 163 cases of successful withdrawal were reported (Orlando et al., 2009). In kidney transplantation, over 200 claimed cases of operational tolerance of over 1 year were reviewed (Orlando et al., 2010). With approximately 28,000 patients undergoing organ transplantation each year, clinicians face a daunting statistic stacked against them.

**FIGURE 1:**
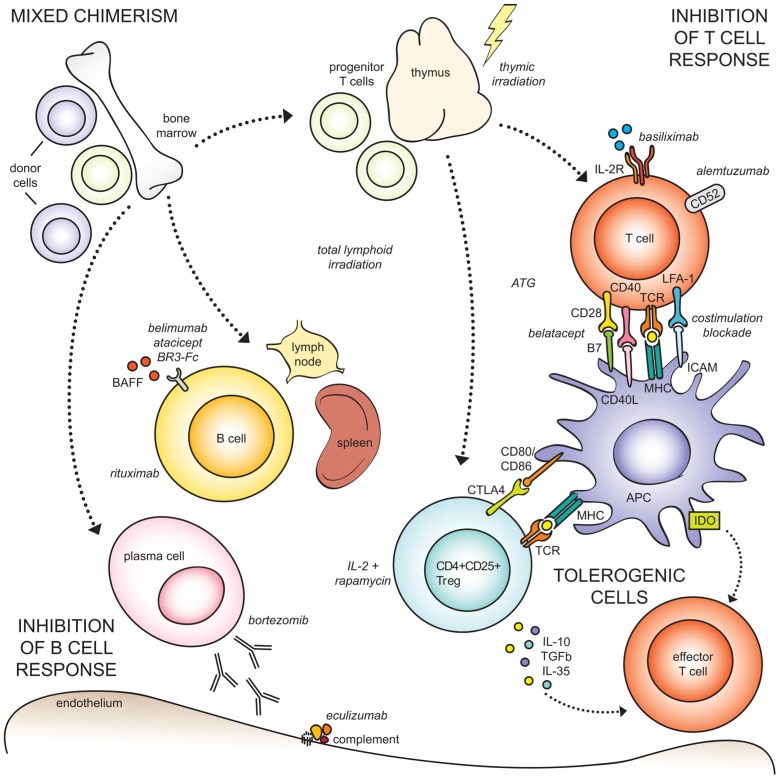
**Approaches to transplant tolerance induction.** (Top left) Mixed chimerism is achieved by infusing donor bone marrow into myelo-conditioned recipients, to establish co-existence of donor and recipient cells in the setting of organ transplantation. The dotted arrows indicate cell types originating from the bone marrow, unrelated to mixed chimerism. (Top right) Allospecific T cell responses can be abrogated through a number of mechanisms, including irradiation, pharmacologic lymphodepletion by ATG or alemtuzumab, suppression of activation by costimulation blockade or IL-2 receptor blockade. (Bottom right) Tolerogenic cell types, including regulatory T cells, macrophages, and mesenchymal stromal cells, can inhibit effector T cells through direct ligation or inhibitory cytokine production. (Bottom left) The humoral response can be suppressed through B cell depletion, and blockade of survival factors (BAFF), plasma cells, and complement.

In pursuit of tolerance, a concerted international effort was made to translate promising basic science findings into clinical practice in transplantation. The US National Institute of Allergy and Infectious Diseases (NIAID) recruited partnerships through tolerance experts in academia, industry, and foundations, and established the US National Institutes of Health sponsored Immune Tolerance Network (ITN) in 1999 (Bluestone et al., 2010). Similarly, the European Commission funded the multinational consortium Reprogramming the Immune System for Establishment of Tolerance (RISET) in 2003. These consortia afforded researchers to evaluate the safety and efficacy of tolerogenic strategies, investigate mechanisms of tolerance, and identify molecular and genetic markers that distinguish the tolerance phenotype. Here, we review traditional and novel approaches to inducing tolerance for organ transplantation (**Figure 1**; **Table 1**). We will discuss within each topic the pre-clinical studies that have or may lead to clinical trials, to focus this topic on the translation of these therapies.

**Table 1:** Strategies for tolerance induction.This table outlines the pharmacologic, biologic, and cellular therapies discussed in this article, categorized byT cell agents, B cell agents, and cellular therapies (including mixed chimerism).

Category	Therapeutic	Mechanism
T cell depletion	Anti-thymocyte globulin (ATG)	Depleting polyclonal antibodies to thymocytes that express multiple target antigens; possible induction of regulatoryT cells
	Alemtuzumab	Depleting mAb to CD52, onT, B, NK cells, some monocytes
Costimulation blockade	Abatacept	CTLA-4 Ig, blockade of CD28:CD80/86 costimulatory pathway
	Belatacept	CTLA-4 Ig, blockade of CD28:CD80/86 costimulatory pathway
	Efalizumab	Blockade of LFA-1:ICAM-1 costimulatory pathway
OtherT cell therapies	Basiliximab	Blockade of CD25 (interleukin 2 receptor α chain)
	Aldesleukin + rapamycin	Interleukin 2 + rapamycin, to increase regulatory T cell proliferation and survival, and stabilize the expression of Forkhead box P3 (FoxP3)
B cell therapeutics	Rituximab	Depleting mAb to CD20
	Belimumab	Blockade of B cell activating factor (BAFF), causing depletion of follicular and alloreactive B cells, decrease in alloantibody response, and promotion of immature/transitional B cell phenotype and a regulatory cytokine environment
	Atacicept	Blockade of BAFF and APRIL
	BR3-Fc	Blockade of BAFF, causing decrease in peripheral, marginal zone, and follicular B cells
	Bortezomib	Proteosome inhibitor, causing apoptosis of mature plasma cells
	Eculizumab	Blockade of complement protein C5, to prevent complement mediated injury due to circulating alloantibody
Cellular therapy	Mixed chimerism	Infusion of donor bone marrow into myoablated/immune-conditioned recipient, to produce coexistence of donor and recipient cells
	Regulatory T cells	Infusion of expanded regulatory T cells, to inhibit inflammatory cytokine production, down-regulate costimulatory and adhesion molecules, promote anergy and cell death, convert effector T cells to a regulatory phenotype, and produce suppressive cytokines IL-10, TGFβ, and IL35
	Regulatory T cells + IL-2	As above, plus the addition of IL-2 to promote Treg survival, development, and expansion
	Dendritic cells	Immunomodulatory effects include their ability to acquire and present antigen, expand and respond to antigen-specific Tregs, constitutively express low levels of MHC and costimulatory molecules, produce high IL-10 and TGFβ and low IL-12, resist activation by danger signals and CD40 ligation, resist killing by natural killer orT cells, and promote apoptosis of effectorT cells
	Macrophages	Immune suppression mediated through the enrichment of CD4^+^ CD25^+^ Foxp3 cells and cell contact- and caspase-dependent depletion of activatedT cells
	Mesenchymal stromal cells	Inhibition of T cell activation and proliferation, potentially due to production of IL-10, NO, and IDO, and suppression of IFNγ and IL-17

## MOLECULE-BASED APPROACHES

### T CELL THERAPIES – DEPLETION

Early attempts at transplantation in humans were fraught with early graft failure due to a robust alloimmune response mediated by activated T cells. We have since learned that the suppression of these alloreactive T cells permits long-term graft survival and, at times, operational tolerance (Starzl et al., 1963; Meier-Kriesche et al., 2004; Womer and Kaplan, 2009). In the 1980s, Strober et al. (1989) observed that some renal transplant patients undergoing total lymphoid irradiation acquired tolerance to their allografts after withdrawal of immunosuppression and demonstrated donor-specific unresponsiveness *in vitro*. Over 30 years later, the concept of eliminating alloreactive T cells upon induction continues to prevail, as T cell depletion remains the most common induction therapy in the U.S (HHS/HRSA/HSB/DOT, 2009). While steroids, calcineurin inhibitors, rapamycin, and mycophenolate mofetil comprise essential components of most immunosuppressive regimens, we will focus our discussion on induction strategies.

Anti-thymocyte globulin (ATG), the oldest depleting agent dating back to the late 1890s, has been a mainstay in induction therapy since the 1960s (Gaber et al., 2010). Due to its potency and markedly heterogeneous target antigen specificities, ATG is particularly useful in high-risk recipients as well as in preventing ischemia-reperfusion injury (Cecka et al., 1993; Shield et al., 1997; Michallet et al., 2003; Bunnapradist and Takemoto, 2005; Chappell et al., 2006; Beiras-Fernandez et al., 2009). ATG has been found to promote regulatory T cells *in vitro* and in murine studies (Lopez et al., 2006; Shimony et al., 2012). The NIAID and ITN are currently conducting a phase II clinical trial using rabbit ATG and rituximab (plus tacrolimus and sirolimus) for tolerance induction in living-donor renal recipients (Markmann, 2011).

Alemtuzumab (Campath-1H, Genzyme), a humanized mAb to CD52 found densely distributed on T and B lymphocytes and natural killer cells (Magliocca and Knechtle, 2006), has been an increasingly popular therapeutic, with three ITN-sponsored trials and over 40 clinical trials registered for liver and kidney transplantation. Ten years ago, we conducted a pilot study of 29 kidney transplant recipients receiving Campath-1H induction and a steroid and calcineurin inhibitor-free maintenance regimen, confirming its efficacy as an induction agent (Knechtle et al., 2003, 2009). When compared to other induction regimens, patients treated with Campath-1H experienced less rejection, especially in patients with delayed graft function, without increased risk of infection or malignancy (Knechtle et al., 2004). Hanaway et al. (2011) in a multicenter, randomized, prospective trial, found that kidney recipients treated with alemtuzumab had significantly reduced early acute rejection rates compared to induction with basiliximab in low-risk and rATG in high-risk patients. As alemtuzumab has been associated with rapid homeostatic proliferation of memory T cells after depletion, increased B cell activating factor (BAFF), and higher rates of alloantibody production and humoral rejection (Knechtle et al., 2003; Pearl et al., 2005; Trzonkowski et al., 2008; Bloom et al., 2009; Thompson et al., 2010), strategic pairing with other immunosuppressive agents may overcome these hurdles. Clinical studies evaluating alemtuzumab in combination therapy with costimulation blockade, regulatory T cell infusion, and donor stem cell transfusion are some of the novel approaches to tolerance induction currently in study.

### T CELL THERAPIES – COSTIMULATION BLOCKADE

Alloreactive T cell activation requires antigen-specific engagement of the T cell receptor with major histocompatibility complex molecules (signal 1), followed by antigen non-specific ligation of a variety of receptor–ligand combinations, or costimulation (signal 2; Jenkins and Schwartz, 1987). Blockade of costimulation effectively prevents T cell activation and allograft rejection (Kirk et al., 1997; Li et al., 1999). While costimulation blockade renders the T cell anergic (Schwartz, 1990), these anergic T cells may express inducible costimulator (ICOS) and play a regulatory role (Vermeiren et al., 2004). In addition, costimulation blockade does not require radical ablation of the immune system by lymphocyte depletion or irradiation, thus shifting the emphasis from induction to maintenance immunosuppression (Larsen et al., 2006).

Costimulatory signals of the CD28:B7 (CD80/86) immunoglobulin superfamily and CD40:CD154 (CD40L) tumor necrosis factor (TNF) family are the most studied and potentially most important activating costimulation pathways. Cytotoxic lympocyte antigen-4 (CTLA-4) shares about 30% homology with CD28, and binds with 10–20-fold higher affinity than CD28 to B7 molecules on the antigen presenting cell (APC). Not only does this potently inhibit the T cell, but also its ligation with APC B7 molecules induces indoleamine 2,3-dioxygenase expression, promoting the suppressive functions in CTLA4^+^ regulatory CD4^+^ cells (Munn et al., 2004). Abatacept (Orencia, Bristol-Myers Squibb) and belatacept (Nulojix, Bristol-Myers Squibb), fusion proteins composed of CTLA-4 and immoglobulin IgG1, have utilized this mechanism to confer potent inhibition of alloreactive T cell responses. Belatacept was developed to increase affinity for CD86; with an increase in affinity by fourfold for CD86 and by twofold for CD80, Belatacept more effectively inhibited T cell activation *in vitro* compared to its predecessor CTLA-4Ig (Larsen et al., 2005). Preclinical studies using CD28:B7 blockade were able to demonstrate prolonged graft survival in non-human primate models of islet transplantation (Adams et al., 2002).

In a randomized, phase III human clinical trial called Belatacept Evaluation of Nephroprotection and Efficacy as First-line Immunosuppression Trial (BENEFIT), recipients of living or standard criteria deceased donors underwent basiliximab induction with mycophenolate mofetil and a steroid taper. Belatacept maintenance, compared to cyclosporine, resulted in superior renal function, cardiovascular and metabolic profiles in the first 2 years (Larsen et al., 2010; Vanrenterghem et al., 2011; Pestana et al., 2012); extension of the trial to recipients of extended criteria donors found similar protective effects on graft function as measured by mean calculated glomerular filtration rate (Pestana et al., 2012). All studies, however, documented increased risk of posttransplant lymphoproliferative disorder in the belatacept-treated arm, compared to the cyclosporine-treated arm.

Activated T cells rapidly upregulate CD154 (CD40L) expression and can bind to CD40, which is constitutively expressed on B cells, dendritic cells (ss), and macrophages (van Kooten and Banchereau, 1997a,b). Blockade of this pathway significantly prolongs allograft survival in non-human primate kidney, heart, skin, peripheral nerve, alloislet, and xenoislet transplantation (Kirk et al., 1997, 1999; Pearson et al., 2002; Xu et al., 2002, 2003; Brenner et al., 2004; Kawai et al., 2004; Adams et al., 2005; Azimzadeh et al., 2006; Hering et al., 2006; Pearl et al., 2007; Aoyagi et al., 2009; Thompson et al., 2011; Badell et al., 2012). Newer antibodies targeting this pathway have avoided platelet activation-induced thromboembolic complications observed with older anti-CD154 mAbs (Koyama et al., 2004). Newer CD40/CD40L blocking agents have yet to be translated to clinical trials.

The lymphocyte function-associated antigen (LFA-1): intracellular adhesion molecule (ICAM) costimulation pathway has also been studied through therapeutic blockade in transplantation. Badell et al. (2010) reported that short-term treatment with LFA-1 prolonged islet allograft in rhesus macaques, and suggested its utility in treating CD28-costimulation blockade-resistant T cell populations. Turgeon et al. (2010) observed that efalizumab (Raptiva, Genentech/Merck Serono) treated patients experienced fewer immunosuppression-related events compared to the standard Edmonton protocol, and also required no additional islet infusions to achieve insulin independence. Efalizumab was withdrawn from the market in 2009 due to a reported increased risk of progressive multifocal leukoencephalopathy (Carson et al., 2009).

### OTHER T CELL THERAPIES

While numerous other surface molecules such as ICOS and very late antigen 4 (VLA-4) have been targeted (Matthews et al., 2003), we will limit discussion here to two trials sponsored by the ITN. In 1999, Shapiro and colleagues presented results from a multicenter, international clinical trial evaluating the Edmonton protocol for islet transplantation, which used interleukin-2 receptor α chain (CD25) blockade for induction (Shapiro et al., 2006). Fifty-eight percent of patients achieved insulin independence, although only 31% of them remained independent after 2 years. While daclizumab (Zenapax, Hoffmann-La Roche), used in the trial, was discontinued in 2009, basiliximab (Simulect, Novartis) remains a popular induction agent. The Kidney Disease: Improving Global Outcomes (KDIGO) group and European Renal Best Practice Advisory Board recommended for all non-high risk kidney transplant recipients to receive IL2R blockade as first line induction therapy (Kasiske et al., 2010).

The ITN is also sponsoring a phase I trial in type I diabetes, using a combination of IL-2 aldesleukin (Proleukin, Prometheus) and rapamycin to arrest islet cell destruction. Animal studies have shown that treatment with IL-2 increases regulatory T cell proliferation and survival (Rabinovitch et al., 2002; Tang et al., 2008). Combination with rapamycin, which stabilizes the expression of Forkhead box P3 (FoxP3) and enhances suppression (Battaglia et al., 2006; Singh et al., 2012), may promote tolerance in these autoimmune and potentially alloimmune settings.

### B CELL THERAPIES

The role of B cells in operational tolerance has yet to be defined. On one hand, an ITN-sponsored collaboration identified a unique B cell signature associated with 25 operationally tolerant renal transplant recipients. Not only did tolerant patients exhibit an increase in total and naïve B cells, but also the majority of genes that were increasingly expressed were B cell-specific, particularly of transitional B cells (Newell et al., 2010). While these transitional B cells could represent a regulatory B cell population based on their increased IL-10 production as discussed by Redfield et al. (2011), no difference in B cell subsets (total, naïve, and transitional cells) or inhibitory cytokines (IL-10 and TGFβ) was detected when compared to healthy controls (Newell et al., 2010).

On the other hand, B cells play a major role in chronic rejection (Kwun and Knechtle, 2009), as donor-specific alloantibodies (DSA) have been causally linked to chronic rejection and long-term graft failure (Eng et al., 2008; Lefaucheur et al., 2008; Terasaki and Cai, 2008; Lee et al., 2009). Patients with pretransplant class I and II DSA have a 10-year graft survival of 30% compared to 72% without (Otten et al., 2012). Donor-specific antibodies, present in approximately 30% of renal transplant candidates on the waiting list (Jordan and Pescovitz, 2006; Jackson and Zachary, 2008) and developing *de novo* post-transplant in 26% of recipients (Terasaki et al., 2006), are a pervasive problem and relevant to the discussion of tolerance induction. While the mechanisms through which B cells may mediate tolerance are unclear, B cells and their therapeutics have certainly emerged as a growing field of interest in transplant immunology.

Long-term allograft acceptance has been achieved by augmenting traditional immunotherapy with B cell depleting antibodies. In cynomolgus macaques, Liu et al. (2007) observed long-term islet allograft survival when rabbit ATG was combined with CD20^+^ B cell-depleting rituximab for induction and rapamycin for maintenance. B cell reconstitution began 100 days after transplantation; long-term survivors exhibited immature and transitional B cells (CD19^+^ CD27-CD38^+^ IgM^+^) in contrast with early rejectors that attained a mature B cell phenotype (CD19^+^ CD27^+^ CD38^+^ IgM^-^). DSA production was inhibited only in the setting of continue rapamycin monotherapy. Compared to cyclosporine alone, treatment with cyclosporine plus rituximab induction (days – 1, 7, 14, and 21) prolonged graft survival, inhibited DSA production, and attenuated chronic rejection in a cynomolgus macaque heart transplantation model (Kelishadi et al., 2010). Kopchaliiska et al. (2009) found that renal transplant patients undergoing B cell depletion for desensitization experienced reconstitution with transitional CD38^+^ B cells and a significant delay in donor HLA-specific CD27^+^ memory B cell repopulation. These studies support that selective use or pairing of B cell depleting agents can generate tolerance promoting B cell phenotypes and eliminate factors leading to chronic rejection. As B cell depletion is inadequate for preventing xeno-specific antibodies (Alwayn et al., 2001) and has had mixed results in desensitization (Ramos et al., 2007; Munoz et al., 2008; Vo et al., 2008; Kozlowski and Andreoni, 2011), further evaluation is needed to optimize its use in transplantation.

Recent studies have used selective targeting of B cell activation and signaling pathways to overcome the problems of DSA and desensitization. BAFF, a member of the TNF family involved in B cell survival, proliferation, and maturation, has been correlated with increased panel reactive antibodies, DSA, B cell repopulation, and C4d^+^ renal allograft rejection (Schneider et al., 1999; Mackay et al., 2003; Xu et al., 2009a,b; Zarkhin et al., 2009). Its blockade using human recombinant mAb belimumab (Benlysta, Human Genome Sciences/GlaxoSmithKline) promoted tolerance in murine cardiac and islet allograft models by (1) depleting follicular and alloreactive B cells, (2) promoting an immature/transitional B cell phenotype, (3) abrogating the alloantibody response, and (4) sustaining a regulatory cytokine environment (Zarkhin et al., 2009; Vivek et al., 2011). The same group evaluated belimumab in a phase II clinical trial for the desensitization of kidney transplant candidates, but recently terminated the study for not reaching efficacy in its primary goals (clinicaltrials.gov ID: NCT01025193). Atacicept (ZymoGenetics/Merck Serono) and BR3-Fc (Briobacept, Genentech/Biogen Idec, discontinued in 2011) are two other BAFF pathway-targeting agents that have demonstrated reduction of alloantibodies and peripheral B cells in non-human primates (Vugmeyster et al., 2006). As Atacicept has failed to show efficacy in clinical trials for rheumatoid arthritis and multiple sclerosis (EMD Serono, 2011; Nanda, 2011), the utility of BAFF/APRIL blockade in human B cell pathology remains to be answered.

Other strategies have focused on plasma cell and complement inhibition for diminishing the humoral response. Bortezomib (Velcade, Millennium), a proteosome inhibitor developed for multiple myeloma and mantle cell lymphoma (Richardson et al., 2003), is an antineoplastic agent causing apoptosis of mature plasma cells. It has been shown to remove alloantibodies and improve allograft function after antibody-mediated rejection (AMR) in kidney, lung, and heart transplant recipients, particularly when combined with plasmapheresis and intravenous immunoglobulin (Patel et al., 2011; Morrow et al., 2012; Stuckey et al., 2012; Sureshkumar et al., 2012), but has had less success in desensitization of renal candidates and late cardiac antibody-mediated rejection (Guthoff et al., 2012; Hodges et al., 2012). Waiser et al. (2012) found that bortezomib was more effective at preserving renal function than rituximab, when given in conjunction with standard therapy for antibody-mediated renal allograft rejection. Currently, three clinical trials are listed for the use of bortezomib in desensitization and clonal deletion of kidney recipients and candidates (clinicaltrials.gov, ID: NCT01349595, NCT00722722, NCT01408797). Eculizumab (Soliris, Alexion) is a recombinant humanized mAb to complement protein C5. Several clinical trials are currently evaluating its efficacy in reducing AMR in DSA + candidates, improving graft function in DSA + recipients, and preventing AMR in ABO blood group incompatible living donor kidney transplantation (clinicaltrials.gov, ID: NCT01327573, NCT01399593, NCT01106027, NCT00670774, NCT01095887).

## CHIMERISM-BASED APPROACHES

Chimerism is the concept that cells of different donor origins can coexist in the same organism, i.e., a form of tolerance. Chimerism itself can be defined into two broad categories: “mixed” or “micro-chimerism” and “full” or “macro-chimerism.” Mixed chimerism is defined as the presence of both donor and recipient cell lineages coexisting in the recipient bone marrow. Full chimerism implies complete elimination of recipient hematopoietic lineages and population of the recipient bone marrow by 100% donor cells (Jankowski and Ildstad, 1997).

As described earlier, Owen was one of the first to describe this finding in the circulating red blood cells of freemartin cattle in which genetically different populations of red blood cells existed in the same animal (Owen, 1945). Its potential application to transplantation was revealed through the work of Medawar and colleagues who found that these same cattle could accept skin grafts from related, but non-identical donors with no immunosuppression (Billingham et al., 1953). Since that time, the idea of hematopoietic chimerism, as a mechanism for tolerance in transplant allograft recipients, has captured the imagination of physicians and researchers working the in the field of organ transplantation.

Practical implementation of this strategy in the clinic has only come to fruition in recent years. The lag in Medawar’s observations and the clinical implementation of his and his colleagues’ findings in solid organ transplant recipients suggests a number of barriers needed to be overcome before clinical application of chimerism could be successful (Jankowski and Ildstad, 1997). The most significant of those barriers is the conditioning of donors and recipients to produce an environment where both donor and host hematopoietic cells can co-exist (Jankowski and Ildstad, 1997; Sachs et al., 2011). In somewhat simplistic terms, a mature host immune system has had time to develop and produce a presumably robust and crowded repertoire of immune cell populations. In order to produce a mixed population of cells, that crowded repertoire must be reduced in size to allow donor hematopoietic cells to exist. Furthermore, recipients must be conditioned to accept these donor cells. Finally, donor cells that could attack the host and cause graft-versus-host disease (GVHD) also need to be eliminated while at the same time preserving the recipient’s ability to produce immune populations that can defend against infections (Jankowski and Ildstad, 1997; Sachs et al., 2011).

These barriers favored a strategy of pursuing mixed chimerism in solid organ transplant recipients, as total marrow ablation associated with full chimerism was thought to be too risky in patients undergoing a semi-elective procedure who would otherwise do well with standard immunosuppression regimens (Sachs et al., 2011). Numerous groups but particularly those of Ilstad and Sachs demonstrated in animal and non-human primate studies that partial irradiation of the recipient bone marrow with peripheral deletion of recipient T cells allowed for the development of both donor and recipient hematopoietic cells and induction of tolerance to donor tissue without the need for full myoablation (Ildstad and Sachs, 1984; Sharabi and Sachs, 1989; Kaufman and Ildstad, 1994; Colson et al., 1995). Mixed chimerism was also found to be beneficial over full chimerism from an infectious risk standpoint both in Ilstad and Sachs’ work as well as in humans undergoing bone marrow transplantation for hematopoietic malignancies (Rayfield and Brent, 1983; Ruedi et al., 1989). While non-myeloablative conditioning only promoted transient mixed chimerism in the HLA-mismatched setting, long-term renal allograft survival was achieved in most patients (Kawai et al., 2011).

Sachs and colleagues took their experimental findings and then went on to implement these strategies in the clinic (Kawai et al., 2008; Spitzer et al., 2011). To date, their group has published two series on induction of mixed chimerism in kidney transplant recipients and subsequent induction of tolerance. Having found that tolerance in chimerism has both a central and peripheral component, their induction strategy now includes thymic irradiation to allow for development of a donor T cell reservoir in these solid organ recipients (Kawai et al., 2008; Sachs et al., 2011; Spitzer et al., 2011).

The results from the aforementioned studies indicate that in both HLA-matched and -mismatched recipients induction of mixed chimerism may be a viable strategy for inducing tolerance in solid organ recipients. To date, of the HLA-matched recipients, seven of eight experienced no episodes of rejection with the single patient with rejection being treated and back on standard immunosuppression. All of these patients also had multiple myeloma so they underwent concomitant bone marrow transplantation. Unfortunately, despite the success of their solid organ transplants, three of the recipients have had recurrence of their multiple myeloma (Sachs et al., 2011; Spitzer et al., 2011). Among the HLA-mismatched patients, one of nine experienced acute rejection, which was effectively treated, and one of nine currently has chronic allograft injury (Kawai et al., 2008; Sachs et al., 2011). The Stanford group recently published their experience of sixteen patients undergoing HLA-matched kidney and hematopoietic cell transplants (Scandling et al., 2012). Conditioning with total lymphoid irradiation and ATG promoted increased proportions of CD4^+^ CD25^+^ regulatory T cells (compared to naïve CD4 T cells) and chimerism in 15 patients. Eight patients had successful withdrawal of immunosuppression for 1–3 years, and only four were unable to withdraw due to recurrent disease or rejection.

These results, though limited, indicate an exciting future for chimerism as a strategy for inducing tolerance in solid organ transplant recipients. They serve as evidence that observations in basic science serve as the basis for new discovery of effective clinical immunosuppressive therapies in the field of transplant surgery.

## OTHER CELL-BASED APPROACHES

### REGULATORY T CELLS

The immune repertoire of experimental animal models and operationally tolerant patients strongly suggests a major role of regulatory T cells (Tregs) in inducing and maintaining tolerance (Graca et al., 2002; Levitsky, 2011). The mechanisms by which these CD4^+^ CD25^+^ T cells exert regulatory control of immune responses are diverse. Upon allorecognition via direct or indirect pathways, Tregs can suppress other T cells through inhibition of cytokine production, down-regulation of costimulatory and adhesion molecules, promotion of anergy and cell death, and conversion of effector T cells to a regulatory phenotype (Wood and Sakaguchi, 2003; O’Garra and Vieira, 2004). A key transcription factor in Treg development and function, Forkhead box protein 3 (Foxp3) has been commonly used to distinguish this population (Hori et al., 2003; Collison et al., 2007), although FoxP3^-^ T cells producing suppressive cytokines IL10 (type I), TGFβ (type 3), and IL35 (type 35) have been identified (Nakamura et al., 2004; Vieira et al., 2004; Collison et al., 2007).

*In vitro* expansion of Tregs has been shown to preserve suppressive function (Levings et al., 2001; Godfrey et al., 2004), thus making it an attractive tolerogenic therapy. Polyclonal expansion using magnetic beads coated with CD3 and CD28 antibodies may yield a several hundred-fold expansion of antigen non-specific Tregs that maintain classic surface and intracellular Treg markers and more importantly their regulatory function (Bluestone, 2005). Hoffmann et al. (2004) documented up to 40,000-fold expansion *in vitro* by repeatedly stimulating with CD3 and CD28 and high dose interleukin 2. While using this technique significantly inhibits graft-versus-host disease (GVHD) as well as allo- and auto-immunity (Taylor et al., 2002; Xia et al., 2006), the inhibitory effect is more pronounced when antigen-specific Tregs are administered (Masteller et al., 2005; Trenado et al., 2006; Nagahama et al., 2007; Zeng et al., 2009; Brennan et al., 2011).

Antigen-specific Tregs can be generated in several ways. Cohen et al. (2002) co-cultured purified CD4^+^ CD25^+^ CD62L^+^ T cells with irradiated splenocytes and observed a significant delay in GVHD development in a murine model. Interestingly, the treated mice later developed severe GVHD, suggesting a limited half-life of these *ex vivo* expanded Tregs. Joffre et al. (2008) observed long-term tolerance in irradiated mice were treated with alloantigen-specific Tregs in bone marrow, and subsequent skin and cardiac allograft models. In a rat liver transplant model, Pu et al. (2007) found that donor-specific splenocyte-stimulated Tregs prolonged graft survival when compared to third party splenocyte stimulated Tregs and freshly isolated syngeneic Tregs. Short-term tacrolimus administration with donor-specific Tregs further enhanced long-term graft acceptance. Yamazaki et al. (2006) observed that dendritic cells were more effective than splenocytes at expanding Tregs and sustaining their Foxp3 expression. Golshayan et al. (2007) used autologous dendritic cells pulsed with an allospecific peptide to promote skin graft tolerance; this approach was later implemented on murine cardiac allografts and paired with short-term rapamycin treatment to achieve indefinite graft survival in three of four mice (Tsang et al., 2009). Peptide-MHC multimers can also be used to create antigen-specific Tregs. Masteller et al. (2005) employed beads coated with recombinant islet peptide mimic-MHC class II plus CD28 antibodies and IL-2; expanded islet peptide mimic-specific Tregs were more efficiently able to suppress autoimmune diabetes in non-obese diabetic mice than polyclonally activated Tregs. Antigen-specific Tregs have also been generated using lentiviral T cell receptor gene transfer into polyclonally expanded cells (Brusko et al., 2010). Finally, Tregs expanded up to 50 million fold by artificial APC s have been shown to maintain suppressor function and reduce GVHD lethality (Hippen et al., 2011). The ability to massively expand functional Tregs in such ways may overcome the challenge of extracting enough circulating Tregs for therapeutic preparation.

*In vivo* expansion of antigen-specific Tregs has also been described in a mouse model (Nishimura et al., 2004). Yamazaki et al. (2003) described the use of antigen-loaded dendritic cells to stimulate CD4^+^ CD25^+^ T cell proliferation *in vivo*, and induce expansion of adoptively transferred CD4^+^ CD25^+^ T cells as well. Walker et al. (2003) found that Tregs deemed anergic based on *in vitro* stimulation assays were capable of proliferating *in vivo* in response to immunization. These studies suggest that therapeutically administered antigen-specific Tregs can continue to be expanded *in vivo*.

The initial clinical trials utilizing Treg immunotherapy for hematopoietic stem cell transplantation (HSCT) have shown promising results (Edinger and Hoffmann, 2011). Brunstein et al. (2011) recently published the University of Minnesota experience, where umbilical cord blood (UCB) derived Tregs were CD3/CD28/IL2 expanded and infused after double UCB transplantation. UCB Tregs were detectable for 14 days, were free of infusion toxicities, and reduced the incidence of severe GVHD. Di Ianni et al. (2011) from the University of Pergia, Italy, observed that co-infusion of Tregs with conventional T cells in the absence of concurrent immunosuppression prevented lethal GVHD and promoted immune reconstitution and protective immunity in 28 patients undergoing HLA-haploidentical HSCT. As interleukin-2 has been found to be critical for Treg survival, development, and expansion (Nelson, 2004; Malek, 2008), it has been administered in clinical trials of autoimmunity and refractory chronic GVHD to augment Treg numbers (Koreth et al., 2011; Saadoun et al., 2011). An important consideration to make of Treg therapy is its cost, with Treg expansion costing $32,000–48,000 per patient (Leslie, 2011).

### TOLEROGENIC DENDRITIC CELLS, MACROPHAGES, AND MESENCHYMAL STROMAL CELLS

Tolerogenic dendritic cells recently have invoked interest in transplantation. Their tolerogenic properties include the ability to acquire and present antigen, expand and respond to antigen-specific Tregs, constitutively express low levels of MHC and costimulatory molecules, produce high IL-10 and TGFβ and low IL-12, resist activation by danger signals and CD40 ligation, resist killing by natural killer or T cells, and promote apoptosis of effector T cells (Thomson et al., 2009). Turnquist et al. (2007) demonstrated indefinite cardiac allograft survival in mice treated with rapamycin-conditioned alloantigen-pulsed dendritic cells. Tregs stimulated by rapamycin-conditioned DCs compared to control Tregs more effectively suppressed antigen-specific T cell proliferation. The regulatory function of DCs mediated by allospecific Treg expansion has also been confirmed in a murine GVHD model (Fujita et al., 2007). To prepare for translation to clinical practice, Boks et al. found that IL-10-generated human tolerogenic DCs were optimal in producing highly suppressive Tregs, compared to conditioning with vitamin D3, dexamathasone, TGFβ, and rapamycin (Boks et al., 2012). They recommended maturing IL-10 DCs with a cocktail of TNFα, IL-1β, and prostaglandin E_2_ (PGE_2_) for optimal migration and stability in pro-inflammatory conditions.

The RISET consortium has supported two clinical trials in the use of transplant acceptance-inducing cell (TAIC) to promote renal allograft survival. The concept of TAIC, an immunoregulatory macrophage, originated from animal models of transplantation and autoimmunity. First, intraportal infusion of rat embryonic stem cell lines in thymus competent rats induced mixed chimerism and allowed permanent acceptance of cardiac allografts (Fandrich et al., 2002). The same group extended this technique of infusing donor-derived TAIC cells to prolong allograft survival in a porcine lung transplant model (Warnecke et al., 2009). In a murine model of inflammatory bowel disease, the infusion of interferon gamma-stimulated monocyte-derived cells (IFNγ-MdC) procured from mouse spleen, blood, and bone marrow reduced inflammation from chronic colitis. These IFNγ-MdC, described as a non-dendritic cell and more mature form of resting macrophages expressing F4/80, CD11, CD86, and PDL-1, mediated their suppressive effects through the enrichment of CD4^+^ CD25^+^ Foxp3 cells and cell contact- and caspase-dependent depletion of activated T cells (Brem-Exner et al., 2008).

In a phase I/II clinical trial, 12 renal transplant recipients underwent postoperative intravenous infusion of macrophages derived from isolated donor splenic monocytes (Hutchinson et al., 2008b). Three of the 12 patients completed their immunosuppression minimization protocol of sequentially withdrawing steroids, sirolimus, and minimizing tacrolimus. Upon confirming the safety of TAIC infusion, a second clinical trial was conducted in five living-related kidney recipients. The induction regimen differed from the first trial, with ATG administered with steroids, tacrolimus, and a preoperative infusion of a greater number of TAICs. Although a higher rate of early acute rejection was observed, three patients were weaned to low-dose tacrolimus monotherapy and one off all immunosuppression for at least 8 months (Hutchinson et al., 2008a). None of the patients in either trial were sensitized to donor antigens using this technique.

Mesenchymal stromal cells (MSCs) have also been evaluated in the transplant setting. Their immunomodulatory properties are several, including their capacity to inhibit T cell activation and proliferation, possibly due to the production of nitric oxide and indoleamine-2,3-dioxygenase (Singer and Caplan, 2011). In addition, upon coculturing with purified immune subpopulations, Aggarwal and Pittenger (2005) described bone marrow-derived MSCs as increasing Treg proportions, decreasing TNFα and IFNγ production by mature DCs, T_H_1 cells, and NK cells, and increasing IL-10, IL-4, and PGE_2_ . Co-infusion of MSCs with donor bone marrow has been shown to enhance mixed chimerism, reverse GVHD, and improve vascularized skin grafts in rats (Aksu et al., 2008). In a rat islet transplantation model, Solari et al. (2009) demonstrated long-term islet allograft survival, normal serum insulin levels, and normoglycemia when autologous MSCs were co-transplanted with marginal islet masses. Promising results from a phase II clinical trial showed that 39 of 55 patients with steroid-resistant, severe acute GVHD responded to MSC therapy and experienced a significant survival benefit (Le Blanc et al., 2008). Phase III randomized, placebo-controlled clinical trials, however, failed to show benefit in the setting of refractory GVHD (Allison, 2009; Ankrum and Karp, 2010).

Recently, MSCs harvested from term fetal membranes have been shown to significantly suppress allogeneic lymphocyte proliferation in mixed lymphocyte reactions, by suppressing IFNγ and IL-17 production and increasing IL-10 production (Karlsson et al., 2012). Duijvestein et al. (2011) found that coadministration with immunosuppressive agents used in inflammatory bowel disease (azathioprine, methotrexate, 6-mercaptopurine, and anti-TNFα antibodies) did not affect MSCs suppressive function *in vitro*, and even had an additive inhibitory effect with some drugs. This suggests that the use of MSCs may be effective in the setting of immunosuppressive drugs used for transplantation as well.

Cell-based approaches to tolerance induction are promising, but further investigation in how these cell populations regulate alloimmune responses is necessary. Moreover, this technology may be limited due to prohibitive costs, availability (with only a few centers capable of amplifying cell populations to sufficient numbers), and issues of standardization and biologics regulation (Bluestone et al., 2007).

## CONCLUSION

Operational tolerance in organ transplant patients continues to be an elusive clinical goal but has stimulated a broad variety of approaches. Research in tolerance has elucidated mechanistic pathways of rejection, T cell regulation, and T cell activation previously unknown. In concert with therapeutic approaches to tolerance, diagnostic assays to identify tolerance and distinguish it from “non-tolerance” are needed, and progress continues in this area relying in part on microarray analysis of tolerant patients. For instance, Li et al. (2012) have identified a small set of 13 genes common to both adult and pediatric liver transplant patients demonstrating operational tolerance. The work by the group of Sanchez-Fueyo continues to publish on biomarkers associated with operationally tolerant liver transplant recipients and their data suggest that both blood and liver tissue gene expression can predict the outcome of immunosuppression withdrawal (Bohne et al., 2012). Interestingly, the genetic signature of tolerance in liver transplantation may differ significantly from that of kidney transplantation for reasons that are unknown at this time (Sagoo et al., 2010). While most clinical work on tolerance focuses on liver transplantation since this organ lends itself best to transplant tolerance, only a miniscule fraction of liver transplant patients appear to have achieved stable tolerance to date, and efforts in this arena need to be conducted under strict clinical guidance in protocols designed to protect the patients’ best interests (Levitsky, 2011). Nevertheless, it would appear likely that as immunologic monitoring evolves into a clinical reality in the coming years, that some patients may benefit from successful withdrawal of immunosuppression while maintaining excellent graft function and intact host defenses.

## Conflict of Interest Statement

The authors declare that the research was conducted in the absence of any commercial or financial relationships that could be construed as a potential conflict of interest.
